# Does Neutrophil to Lymphocyte Ratio Have a Role in Identifying Cytokeratin 19-Expressing Hepatocellular Carcinoma?

**DOI:** 10.3390/jpm11111078

**Published:** 2021-10-24

**Authors:** Chao-Wei Lee, Sey-En Lin, Ming-Chin Yu, Hao-Wei Kou, Cheng-Han Lee, Tony Kuo, Kuan-Chieh Lee, Hsin-I Tsai

**Affiliations:** 1 Division of General Surgery, Department of Surgery, Chang Gung Memorial Hospital, Linkou Medical Center, Guishan, Taoyuan 333, Taiwan; alanchaoweilee@hotmail.com (C.-W.L.); mingchin2000@gmail.com (M.-C.Y.); jeffreykou0417@gmail.com (H.-W.K.); 2 College of Medicine, Chang Gung University, Guishan, Taoyuan 333, Taiwan; b9102011@cloud.cgmh.org.tw (C.-H.L.); B9302028@cgmh.org.tw (T.K.); guanjie0826@gmail.com (K.-C.L.); 3 Graduate Institute of Clinical Medical Sciences, Chang Gung University, Guishan, Taoyuan 333, Taiwan; 4 Department of Anatomic Pathology, New Taipei Municipal Tucheng Hospital (Built and Operated by Chang Gung Medical Foundation), Tucheng, New Taipei City 236017, Taiwan; linse@cgmh.org.tw; 5 Department of Surgery, New Taipei Municipal Tu-Cheng Hospital (Built and Operated by Chang Gung Medical Foundation), Tucheng, New Taipei City 236017, Taiwan; 6 Department of Gastroenterology and Hepatology, Chang Gung Memorial Hospital, Linkou Medical Center, Guishan, Taoyuan 333, Taiwan; 7 Department of Anesthesiology, Chang Gung Memorial Hospital, Linkou Medical Center, Guishan, Taoyuan 333, Taiwan

**Keywords:** neutrophil to lymphocyte ratio, NLR, hepatocellular carcinoma, hepatoma, cytokeratin 19 (CK19)

## Abstract

Background: Cytokeratin 19-positive (CK19(+)) hepatocellular carcinomas (HCC) are generally associated with poor prognosis after hepatectomy. It is typically detected from postoperative immunochemistry. We have analyzed several clinically available biomarkers, in particular, neutrophil to lymphocyte ratio (NLR) and aim to develop a panel of biomarkers in identifying CK19 expression in (HCC) preoperatively. Methods: We retrospectively reviewed 36 HCC patients who underwent liver resections during January 2017 to March 2018 in Chang Gung Memorial Hospital. Patients were grouped based on the status of CK19 expression and their baseline characteristics, perioperative and oncologic outcomes were compared. Novel biomarkers including NLR, alpha-fetoprotein (AFP), carcinoembryonic antigen (CEA) and uric acid were analyzed and correlated with CK19 expression. Results: NLR is highly associated with CK19 expression. NLR alone gave an AUROC of 0.728 (*p*-value = 0.043), higher than AFP, CEA or tumor size alone. NLR when combined with AFP, CEA and uric acid, gave an AUROC as high as 0.933 (*p*-value = 0.004). Conclusion: The current study demonstrated the predictive capability of NLR in combination with AFP, CEA and uric acid for CK19 expression in HCC patients preoperatively. Further prospective, large-scale studies are warranted to validate our findings.

## 1. Introduction

As the fifth most common malignancy and the third leading cause of malignancy-related mortality, the incidence of hepatocellular carcinoma (HCC) is still on the rise [1,2]. Despite an array of curative and palliative treatments involving surgical resection, liver transplantation, radiofrequency ablation (RFA), transarterial chemoembolization (TACE) and systemic targeted therapy, the five-year survival rate remains low with a five-year tumor recurrence rate of approximately 70% [3,4]. Based on molecular features, HCCs are grouped into non-proliferative and proliferative subtypes, both of which is characterized by the presence of cytokeratin 19 (CK19). Clinically, CK19 is typically detected from postoperative immunochemistry. CK19-positive HCCs not only display more aggressive behavior in association with invasion and angiogenesis, but also worse prognosis, early tumor recurrence and worse overall survival as compared to CK19-negative HCC [5,6,7]. Recently, CYFRA 21-1, a soluble fragment of CK19, has been detected in peripheral circulation in various malignancies including HCCs [8,9,10,11]. Although CYFRA 21-1 appears to be a sensitive biomarker for CK19 expression in HCCs with a predictive power of 0.81 [11,12], routine laboratory test of preoperative serum CYFRA 21-1 may be limited, as CK19 expression is only present in approximately 10–30% of HCC patients. Recently, neutrophil to lymphocyte ratio (NLR) has been widely studied for post-therapeutic recurrence and survival in HCC [13,14,15]. Lymphocytes are involved in cell-mediated anti-tumor immune responses [16] and NLR has been found to be independently associated with survival in HCC patients [17]. The aims of the study were to examine the relationship between NLR and CK19 expression in HCCs and to develop a panel of biomarkers in the prediction of CK19 expression. 

## 2. Materials and Methods

### 2.1. Patients

Medical records of patients spanning from January 2017 to March 2018 with histological proven primary HCC from the Cancer Registry of the Cancer Center, Chang Gung Memorial Hospital (CGMH), Linkou, Taiwan were retrospectively reviewed. The study was approved by the CGMH Institutional Review Boards (IRB 202002256B0). Patients undergoing curative hepatectomy by the same surgical team were eligible for the study and their clinico-pathological data were retrieved from the prospectively collected database. Age, gender, cigarette smoking, alcohol consumption, viral infection, preoperative alpha-fetoprotein (AFP), carcinoembryonic antigen (CEA), Child-Pugh classification, tumor size, encapsulation, staging histological grade and resection margin were collected for analysis. Tumor staging was based on the 8th edition of American Joint Committee on Cancer Tumor-node-metastasis (AJCC TNM) staging system for HCC.

### 2.2. Immunohistochemistry (IHC)

Formalin-fixed and parafiin-embedded resection specimens were sectioned to 4 µm in thickness and deparaffinized, rehydrated and processed for antigen retrieval. Incubated at room temperature with diluted (1:100) monoclonal antibody to CK19 (Abcam, San Francisco, CA, USA) for 1 h, the slides were initially washed in phosphate buffered saline for three times, further incubated with a horse reddish peroxidase conjugated antibody (ZYMED, San Francisco, CA, USA) for 10 min and then developed by treatment with 3,3-diamniobenzideine (Dako North America, Inc., Carpinteria, CA, USA) for another 10 min. Experienced pathologist independent to the study and naïve to the patient characteristics and outcomes determined the results of immunohistochemical staining under microscope. A positive CK19 expression was defined as ≥5% of tumor cells stained positive for CK19 [18].

### 2.3. Statistical Analysis

The statistical analysis was performed with IBM SPSS Statistics 21 (IBM Corporation, Software Group, Somers, NY USA). Categorical data was analyzed using Fisher’s exact test and Pearson’s χ^2^ test and quantitative variables were analyzed by student’s *t* test for normally distributed data and the Mann-Whitney U test for non-normally distributed ones. For the prediction of CK19 expression, logistic regression and receiver operating characteristic of variable factors was performed. For all statistical tests, *p*-value < 0.05 was considered statistically significant.

## 3. Results

### 3.1. Patient Demographics

**Figure 1:**
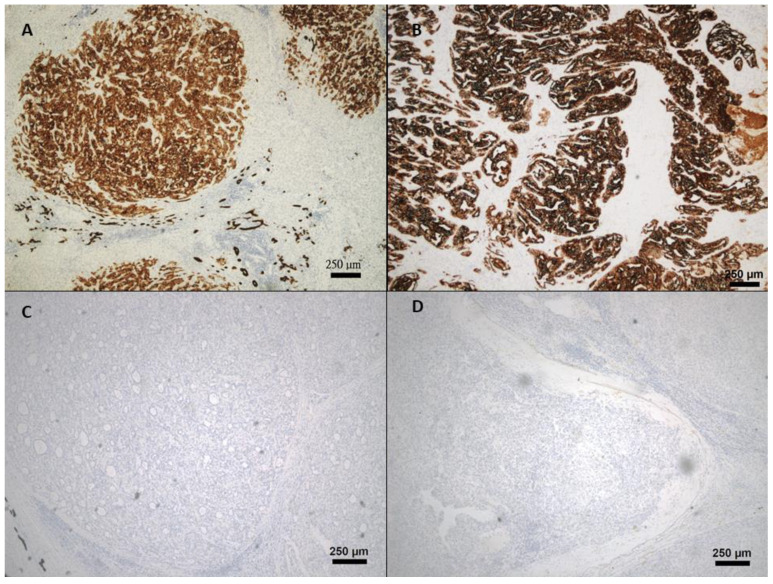
Immunohistochemical microphotograph of HCC with CK19 expression. (**A**,**B**): CK19(+) HCC; (**C**,**D**): CK19 (-) HCC. Magnifications, ×40.

The demographic and clinical characteristics of patients with HCC undergoing hepatectomy were summarized in Table 1. A total of 36 patients were enrolled with a mean age of diagnosis 63.6 ± 1.6 years and a majority of males (*n* = 26, 72.2%). All patients were Child-Pugh A liver cirrhosis. 52.8% of patients were hepatitis B virus (HBV)) carriers, 30.6% patients had chronic hepatitis C virus (HCV)) infection and 16.7% had chronic alcohol consumption. The mean indocyanine green retention at 15 min (ICG-15) was 10.4 ± 0.88% and NLR of 2.2 ± 0.21. The mean preoperative AFP and CEA levels were 4161.7 ± 3782.62 ng/mL and 6.2 ± 4.07 ng/mL, respectively. As for pathological variables, the majority of patients had a tumor size less than 5 cm (n = 24, 66.7%) with a mean of 4.7 ± 0.53 cm, of all, 80.6% were encapsulated. Tumor rupture was found in only three (8.6%) cases. Vascular invasion was found in 11 (30.6%) of all participants and three (8.3%) tumors had daughter nodules. All patients achieved complete tumor resections (negative margin). 44.4% had histology-proven liver cirrhosis and 30% had tumor necrosis. 44.4% and 55.6% patients had Edmonson and Steiner grade I/II and III/IV, respectively. CK19 expression was positive in 9 (25%) tumors (Figure 1).

### 3.2. Analysis of Biomarkers Predicting CK19 Expression

Table 2 summarized the relationship between clinicopathological variables and CK19. Patient age, gender, Child-Pugh classification, ICG-15, tumor size, tumor rupture, tumor encapsulation and daughter nodules were not related to CK19 expression in HCC; however, the occurrence of lymph node metastasis after operation was significantly more prevalent in CK19(+) patients. Among other parameters, although statistically insignificant, the levels of triglyceride, cholesterol and uric acid were found to be consistently lower in CK19 (+) patients. Of note was that NLR was found to be significantly elevated in CK19 (+) patients (3.0 ± 0.67 vs. 1.9 ± 0.13) with a *p*-value = 0.043.

### 3.3. Performance of Biomarkers in Predicting CK19 Expression in HCC

**Figure 2:**
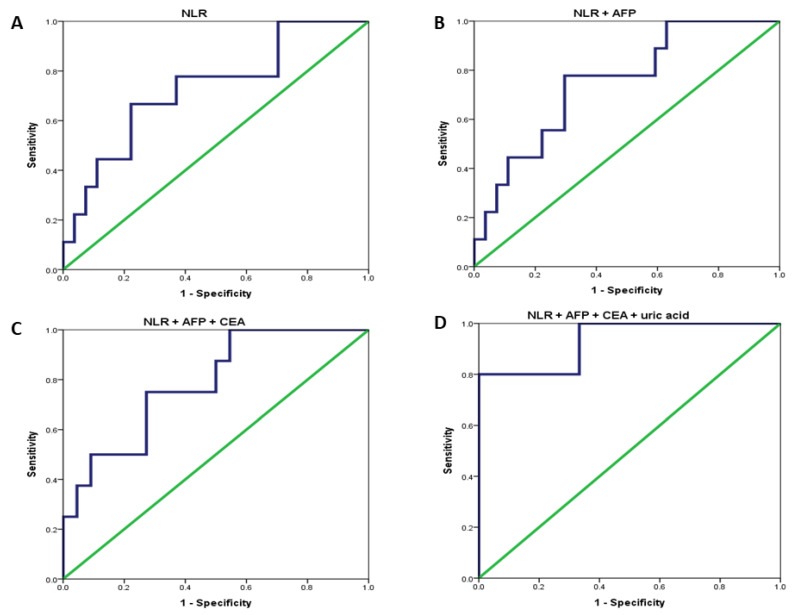
Performance of Biomarkers in Predicting CK19 expression in HCC illustrated as Area Under the ROC (AUROC). (**A**) NLR; (**B**) NLR + AFP; (**C**) NLR + AFP + CEA; (**D**) NLR + AFP + CEA + uric acid.

To predict the possibility of CK19 expression in HCC pre-surgically, receiver operating characteristic (ROC) curves were employed and various clinical parameters were analyzed as shown in Table 3 and Figure 2. In predicting CK(+) status in HCC patients, the area under the ROC curves (AUROC) of AFP, CEA and NLR were 0.683, 0.670 and 0.728, respectively, with no statistical significance. NLR in combination with AFP or CEA alone and altogether increased the AUROCs to 0.749 (*p*-value = 0.027), 0.756 (*p*-value = 0.035) and 0.784 (*p*-value = 0.019), respectively. Interestingly, an AUROC of 0.933 with a *p*-value of 0.004 was achieved when AFP, CEA and uric acid were altogether incorporated into analysis.

## 4. Discussion

Despite an improvement in current therapeutic strategies in HCC, morbidity and mortality rates of HCC are still increasing. In clinical practice, clinicians rely on tumor number, tumor size and macro-vascular invasion in addition to several biomarkers such as AFP, CEA and CK19 to predict HCC prognosis. Clinically, CK19 is typically detected from the postoperative immunochemistry. CK19 (+) HCCs are not only associated with early tumor recurrence and poor overall survival after hepatectomy or liver transplantation, but are also resistant to chemotherapy and local treatment as compared to CK19 (-) HCCs [6,19,20,21]. Belonging to a family of keratins, CK are normally expressed in the lining of the gastroenteropancreatic and hepatobiliary tract. CK immunohistochemistry is often performed in the evaluation of various tumors [22,23,24]. Among those, CK19 is of great interest in the study of HCCs. During the embryonic development, CK19 is initially detected in the primitive hepatic progenitor cells, which then differentiate into hepatocytes or biliary epithelial cells in fetal development [25]. As hepatocytes mature, CK19 is no longer detectable in hepatocytes but only present in biliary epithelial cells. However, when hepatocytes have insults secondary to chronic liver diseases, the normal liver cells become activated and transformed into progenitor-like cells, which is when CK19 re-emerges on hepatocytes [26,27]. Considering the distinct invasive characteristics of CK19 (+) HCCs, this subtype of HCCs should be detected early in guiding individualized therapy in clinical context. 

Recently, researchers have suggested that neutrophil to lymphocyte ratio (NLR) is associated with tumor response and progression-free survival in HCCs after treatment [28,29]. Particularly, lower pre-treatment NLR had better recurrence-free survival rates [30]. The exact molecular mechanisms through which the elevated NLR is associated with poor HCC outcomes remain unidentified. Neutrophilia may inhibit the cytolytic activity of immune cells while augmenting angiogenesis progression in the peritumoral stroma. Neutrophils in the intratumoral regions of HCC also increase the autophagic activity, which sustains poor survival and the pro-tumorigenic effects [31,32]. On the other hand, lymphocytes are involved in cell-mediated anti-tumor immune response [16,33]. Lower levels of lymphocytic infiltration have been shown as a predictor of poor prognosis for colorectal liver metastases and HCC [34,35]. 

Consistent with the literature, we have found that NLR is highly associated with CK19 expression, which may contribute to its worse survival. We have also examined the performance of commonly used biomarkers such as AFP, CEA and other clinical parameters in predicting CK19 expression in HCCs. The current study has demonstrated that NLR alone gave an AUROC of 0.728, higher than AFP, CEA or tumor size alone. Moreover, NLR, when combined with AFP, CEA and uric acid, gave an AUROC as high as 0.933. Uric acid has recently been identified as a predictor for liver function after living donor liver transplantation [36]. Uric acid has been known for its antioxidant activity in various diseases and the liver is the major site of uric acid production. In severe hepatocellular injury, the production of uric acid is greatly reduced. Our finding indicates the possibility of a panel incorporating NLR and uric acid as readily available, reliable and inexpensive biomarkers for making clinical decisions regarding HCC treatment.

Despite remarkable results, the current study still has limitations. First, the sample size was limited, especially of those with CK 19 expression. Second, as a retrospective study, selection bias is inevitable. Third, although our panel revealed the predictive capability for CK19 expression, the exact mechanisms remain undetermined. Fourth, the lack of an external validation cohort also rendered the current study less robust. Therefore, future prospective studies of a larger population size are warranted to validate and explain our findings. 

## 5. Conclusions

As one of the leading causes of malignancy-related mortality, the poor prognosis of HCC is largely determined by the presence of CK19 expression. We have developed a panel of biomarkers incorporating NLR and other commonly tested parameters, namely AFP, CEA and uric acid as a readily available and inexpensive method in predicting CK19 expression. Such a finding is believed to aid surgeons preoperatively in determining the characteristics of HCC and the prognosis for patients. Due to limited sample size and confounding factors, future larger scale studies are warranted to validate our findings.

## Figures and Tables

**Table 1:** Demographic data of patients with hepatocellular carcinoma undergoing hepatectomy (n = 36).

Variables	No. (%)	Variables	Mean ± SE ^e^/Median (IQR) ^f^
Age (≤65 year-old)	19 (52.8)	Age (year-old)	63.6 ± 1.60
Gender (Male)	26 (72.2)	ICG-15 (%)	10.4 ± 0.88
Comorbidity		Preoperative α-fetoprotein (ng/mL)	18.05 (194.5)
Diabetes Mellitus (Yes)	12 (33.3)	Preoperative CEA ^d^ (ng/mL)	1.99 (2.22)
Hypertension (Yes)	22 (61.1)	Tumor size (cm)	4.7 ± 0.53
Smoking (Yes)	12 (33.3)	Neutrophil to lymphocyte ratio	1.93 (1.0)
Alcohol (Yes)	6 (16.7)	**Variables**	**No. (%)**
Child-Pugh Classification (A)	36 (100)	Vascular invasion (Yes)	11 (30.6)
Preoperative α-fetoprotein (>200 ng/mL)	9 (25.0)	Daughter nodules (Yes)	3 (8.3)
ICG-15 ^a^ (≤10%)	19 (52.8)	Resection margin (negative)	36 (100)
Tumor size (>5 cm)	12 (33.3)	Edmonson and Steiner grade (I/II vs. III/IV)	16(44.4) vs. 20(55.6)
HBV infection ^b^	23 (63.9)	Liver cirrhosis (Yes)	16 (44.4)
HCV infection ^c^	11 (30.6)	Tumor necrosis (Yes)	11 (30.6)
Tumor encapsulation (Yes)	29 (80.6)	AJCC stage (I/II/III/IV) ^g^	20(55.6)/9(25.0)/6(16.7)/1(2.8)
Tumor rupture (Yes)	3 (8.3)	CK19 expression (Yes)	9 (25.0)

^a^ indocyanine green retention at 15 min. ^b^ hepatitis B virus. ^c^ hepatitis C virus. ^d^ carcinoembryonic antigen. ^e^ standard error of mean. ^f^ interquartile range. ^g^ American Joint Committee on Cancer.

**Table 2:** The relationship between clinicopathological variables and CK19 expression in hepatocellular carcinoma.

Hepatocellular Carcinoma, *n* = 36							
	CK19 ^a^			CK19 ^a^			
	Negative (%) ^b^	Positive (%) ^c^	*p* Value		Negative (%) ^b^	Positive (%) ^c^	*p* Value
Age(yr)			0.706	Vascular invasion			0.409
>65	12 (44.4)	5 (55.6)		Yes	7 (25.9)	4 (44.4)	
Gender			1.000	Tumor rupture			0.558
Male	19 (70.4)	7 (77.8)		Yes	3 (11.1)	0 (0)	
Child-Pugh classification			1.000	Daughter nodules			0.558
A	27 (100)	9 (100)		Yes	3 (11.1)	0 (0)	
ICG-15 (%)			0.118	Resection margin			N/A ^d^
≤10	10 (40.0)	7 (77.8)		Positive	0 (0)	0 (0)	
α-fetoprotein (ng/mL)			1.000	Edmonson Grade			0.419
>200	7 (25.9)	2 (22.2)		III/IV	14 (51.9)	6 (75.0)	
Size (cm)			0.443	Tumor necrosis			0.690
>5	8 (29.6)	4 (44.4)		Yes	9 (33.3)	2 (22.2)	
Lymph node metastasis			0.002	Tumor size (cm) ^e^	4.5 ± 0.62	5.2 ± 1.05	0.472
Yes	0 (0)	4 (44.4)		α-fetoprotein (ng/mL) ^f^	8.9 (219.6)	68.9 (179.5)	0.109
Lung metastasis			1.000	PLR ^eg^	97.2 ± 7.11	114.2 ± 9.32	0.217
Yes	2 (7.4)	1 (11.1)		NLR ^fh^	1.81 (0.73)	2.32 (1.48)	0.043
Intrahepatic metastasis				CEA ^fi^ (ng/mL)	1.58 (2.53)	2.45(1.015)	0.599
Yes	8 (29.6)	3 (33.3)	1.000	Triglyceride (mg/dL) ^f^	115 (83.5)	49 (81)	0.080
Encapsulation			1.000	Cholesterol (mg/dL) ^e^	165.5 ± 7.71	158.3 ± 18.8	0.693
Yes	22 (81.5)	7 (77.8)		Uric acid (mg/dL) ^e^	6.1 ± 0.39	4.3 ± 0.70	0.058

^a^ Immunohistochemical staining of primary liver tumor specimen for CK19; ^b^ percentage within CK19 (-) HCC; ^c^ percentage within CK19(+) HCC; ^d^ not applicable; ^e^ mean ± standard error of mean; ^f^ median (interquartile range); ^g^ platelet to lymphocyte ratio; ^h^ neutrophil to lymphocyte ratio; ^i^ carcinoembryonic antigen.

**Table 3:** Area under the ROC curve (AUROC) in predicting CK19 expression in hepatocellular carcinoma (n = 36).

Variables	Prediction of CK19 Expression			
	AUROC	Cutoff Value	Sensitivity/Specificity(%)	*p* Value
Neutrophil to lymphocyte ratio (NLR)	0.728	1.976	77.8/63.0	0.043
α-fetoprotein (AFP, ng/mL)	0.683	20.85	77.8/63.0	0.104
CEA ^a^ (ng/mL)	0.670	1.865	100/59.1	0.159
Tumor size (cm)	0.582	3.650	55.6/55.6	0.465
Uric acid (mg/dL)	0.750	4.50	86.4/50.0	0.065
Triglyceride (mg/dL)	0.767	53.5	94.460.0	0.074
NLR + AFP	0.749			0.027
NLR + CEA	0.756			0.035
NLR + AFP + CEA	0.784			0.019
NLR + AFP + CEA + uric acid	0.933			0.004

^a^ carcinoembryonic antigen.

## Data Availability

All data generated or analyzed during the study are included in this published article. Raw data may be requested from the authors with the permission of the institution.
